# Potential of niacin skin flush response in adolescent depression identification and severity assessment: a case-control study

**DOI:** 10.1186/s12888-024-05728-w

**Published:** 2024-04-17

**Authors:** Jie Feng, Wenjiao Min, Dandan Wang, Jing Yuan, Junming Chen, Lisha Chen, Wei Chen, Meng Zhao, Jia Cheng, Chunling Wan, Bo Zhou, Yulan Huang, Yaoyin Zhang

**Affiliations:** 1 Department of Psychosomatics, School of Medicine, Sichuan Provincial Center for Mental Health, Sichuan Provincial People’s Hospital, University of Electronic Science and Technology of China, No. 32, West second Section, 1st Ring Road, 610041 Chengdu, Sichuan China; 2 Sichuan Provincial Center for Mental Health, Sichuan Academy of Medical Science & Sichuan Provincial People’s Hospital, No. 33, Section 2, Furong Avenue, Wenjiang District, 611135 Chengdu, Sichuan China; 3 Key Laboratory of Psychosomatic Medicine, Chinese Academy of Medical Sciences, Chengdu, China; 4 Bio-X Institutes, Key Laboratory for the Genetics of Developmental and Neuropsychiatric Disorders, Ministry of Education, Shanghai Jiao To ng University, Shanghai, China; 5 School of Nursing, Chengdu Medical College, Chengdu, China

**Keywords:** Niacin skin flush response, Adolescent depressive disorder, Behavioral and emotional disorders typically emerging in childhood and adolescence, Biomarker, Precision diagnosis

## Abstract

**Background:**

The diagnosis of adolescent Depressive Disorder (DD) lacks specific biomarkers, posing significant challenges. This study investigates the potential of Niacin Skin Flush Response (NSFR) as a biomarker for identifying and assessing the severity of adolescent Depressive Disorder, as well as distinguishing it from Behavioral and Emotional Disorders typically emerging in childhood and adolescence(BED).

**Methods:**

In a case-control study involving 196 adolescents, including 128 Depressive Disorder, 32 Behavioral and Emotional Disorders, and 36 healthy controls (HCs), NSFR was assessed. Depressive symptoms were measured using the Patient Health Questionnaire-9 (PHQ-9) and anxious symptoms with the Generalized Anxiety Disorder 7-item scale (GAD-7). Pearson correlation analysis determined the relationships between NSFR and the severity of depression in DD patients. Receiver Operating Characteristic (ROC) was used to identify DD from BED integrating NSFR data with clinical symptom measures.

**Results:**

The adolescent Depressive Disorder group exhibited a higher rate of severe blunted NSFR (21.4%) compared to BED (12.5%) and HC ( 8.3%). Adolescent Depressive Disorder with psychotic symptoms showed a significant increase in blunted NSFR (*p* = 0.016). NSFR had negative correlations with depressive (*r* = -0.240, *p* = 0.006) and anxious (*r* = -0.2, *p* = 0.023) symptoms in adolescent Depressive Disorder. Integrating NSFR with three clinical scales improved the differentiation between adolescent Depressive Disorder and BED (AUC increased from 0.694 to 0.712).

**Conclusion:**

The NSFR demonstrates potential as an objective biomarker for adolescent Depressive Disorder, aiding in screening, assessing severity, and enhancing insights into its pathophysiology and diagnostic precision.

## Introduction

Major Depressive Disorder (MDD) presents a pressing challenge in adolescent mental health, with prevalence estimates for adolescent Depressive disorder (DD) as high as 25.2% [1]. This is particularly concerning in China, where up to 40.1% of high school students show depressive symptoms, and 8% exhibit severe depression [2, 3]. Moreover, depression significantly contributes to adolescent suicide [4]. It is also linked to various adverse outcomes, such as academic and social difficulties, substance abuse, and self-harm [5].

The etiology of Adolescent Depressive Disorder (DD, ICD-10 F32) involves complex biological and psychosocial influences, leading to varied clinical presentations and treatment outcomes [6, 7]. However, severe cases often go unrecognized or inadequately treated, with less than half diagnosed before adulthood [8].Current diagnostic methods, including structured interviews and scales, are inefficient and lack objective precision. Additionally, Behavioral and Emotional Disorders (BED, ICD-10 F98.9) typically emerging in childhood and adolescence present a spectrum of psychological symptoms overlapping with DD, challenging accurate diagnosis under ICD-10 guidelines. BED encompass a spectrum of emotional and behavioral challenges. This includes cases where children experience emotional distress, but do not meet the criteria for severe Depressive Disorder (DD), yet exhibit recurrent emotional issues and self-harm. BED also includes individuals with mood fluctuations and irritability that do not fit the diagnosis of bipolar disorder, as well as those with transient emotional downturns due to external factors, which improve once these influences subside [9, 10]. This symptom overlap between BED and DD complicates differential diagnosis, underscoring the urgent need for unique biomarkers for accurate disorder identification [11].

Prior research highlights the potential of the Niacin Skin Flush Response (NSFR) in delineating adult patients with Schizophrenia and Affective Disorders from healthy controls, demonstrating a positive predictive value of 93.66% [12]. The Niacin Skin Flush Response (NSFR) displays a delayed response in adult depression, showing an inverse correlation with symptom severity as noted in previous studies [13, 14].Similar patterns have been noted in pediatric psychiatric conditions [15]. Notably, Jinfeng Wang et al. reported attenuated NSFR in adolescent depression [16], underscoring its potential as a marker for Depressive Disorder (DD). However, further exploration is needed regarding the role of NSFR in diagnosing and differentiating Adolescent Depressive Disorder.

The Niacin Skin Flush Response (NSFR), associated with inflammation and oxidative stress, may indicate disruptions in arachidonic acid metabolism, potentially pointing to lipid imbalances in the brain that could be significant for understanding depressive disorders [16, 17]. While existing biomarkers examine monoamine neurotransmission, immune inflammation, neuroplasticity, and neuroendocrine functions, focusing on protein dysfunction, NSFR stands out due to its specific metabolic insights [18, 19]. Its non-invasiveness, ease of administration, and repeatability suggest it could offer valuable insights, particularly in adolescent depression [19].

Depressive Disorder (DD) with psychotic features(ICD-10 F32.3) is identified as a distinct subtype, highlighting the clinical need to distinguish between depressive disorders with varying presentations. Evidence shows the Niacin Skin Flush Response (NSFR) can discern adult schizophrenia from depression, with varying NSFR patterns correlated to psychiatric conditions [12, 20]. This study probes NSFR’s viability as a biomarker to differentiate DD with and without psychotic symptoms((ICD-10 F32.0-F32.2) ), aiming to clarify DD subclassifications.

This study aims to evaluate the Niacin Skin Flush Response (NSFR) as a biological marker for adolescent Depressive Disorder (DD), with the objective of improving screening accuracy, assessing severity, classifying subgroups, and differentiating DD from BED.

## Material and method

### Participants

Our study conducted from January to October 2023, at Sichuan Provincial People’s Hospital, Chengdu, China. **Inclusion criteria**:(1)Adolescents aged 12–18 years. (2)Adolescents were diagnosed with Depressive Disorder (ICD-10 F32) and Behavioral and Emotional Disorders (BED, ICD-10 F98.9) typically emerging in childhood and adolescence according to International Classification of Diseases, 10th (ICD-10). (3) Diagnosis confirmed via the Composite International Diagnostic Interview (CIDI) by two psychiatrists. (4)The Patient Health Questionnaire-9 (PHQ-9) Score ≥ 10. **Exclusion criteria**: Any participant with neurological diseases, brain injury, severe skin diseases, or immune diseases was excluded from this study. Those who used anti-inflammatory drugs within 2 weeks were also excluded.

The experimental group was assembled from a cohort of 208 adolescents admitted to the Child and Adolescent Psychiatry Inpatient Department, employing a randomized selection method on Tuesdays and Thursdays of each week. This group ultimately included 160 patients, comprising 128 individuals diagnosed with Depressive Disorder (ICD-10 F32) and 32 with Behavioral and Emotional Disorders typically emerging in childhood and adolescence (BED) (ICD10 F98.9). The Depressive Disorder was further stratified by the presence (ICD-10 F32.3) or absence (ICD-10 F32.0-F32.2) of psychotic symptoms. We excluded those 48 with Bipolar Disorder, Neuro-developmental disorders, Adjustment Disorder, Schizophrenia, Organic Mood Affective Disorder, Obsessive-Compulsive Disorder, PTSD, or Dissociative disorders. For healthy controls (HCs), 50 high school students were initially considered through targeted advertisements, with 36 ultimately selected post PHQ-9 screening and CIDI confirmation, ensuring no history of mental illness.

Before participation, comprehensive information about the study and testing procedures was provided to all participants. Their parents or legal guardians also received this information. Informed consents were then obtained from all participants and their legal guardians.

### Assessment of niacin skin flush response (NSFR)

#### Methodology

The Niacin Skin Flush Response (NSFR) of participants was evaluated using the Brain Aid Skin Niacin Response Test Instrument TY-AraSnap-H100, manufactured by Shanghai Tianyin Biological Technology Ltd., Shanghai, China. This instrument employs a transdermal delivery system involving a patch. The patch administers an aqueous solution of methyl nicotinate (AMN), which has a chemical formula of C7H7NO2 and a purity of 99%, as provided by Sigma-Aldrich. Furthermore, the patch’s unique design features six circular apertures that penetrate its sponge and adhesive layers. These apertures expose the filter paper layer beneath, which is specifically designed to facilitate the delivery of AMN.

A precise volume of the AMN solution was applied to the patch’s apertures. The solution came in varying concentrations: 60 mM, 20 mM, 6.67 mM, 2.22 mM, 0.74 mM, and 0.25 mM. After applying the solution, the patch was affixed to the volar aspect of the participant’s left forearm. It remained there for a standardized duration of one minute. Following this, the patch was carefully removed. A time-lapse photographic record was then established. Images were captured at ten-second intervals over a ten-minute period. These images documented the erythema response. From this process, a set of 60 photographs was obtained for each participant.

An initial photograph served as a baseline for comparative analysis. The instrument’s software then quantified the erythema at 59 time points. These points were under six different AMN concentrations. This process generated 354 measurements for each participant. The software analyzed the erythema surface area responses. It focused on the first quartile of AMN concentrations. This analysis helped determine the overall NSFR magnitude.

We lacked data specific to adolescents. Therefore, we used an adult-referenced model from the BrainAid database to evaluate adolescent NSFR. This database is accessible at http://brainaid.sjtu.edu.cn. The model is based on adult depression-related niacin patterns. It helped us classify adolescents’ niacin responses as either ‘blunted’ or ‘normal.’ We divided the ‘blunted’ responses into three categories: mild, moderate, and severe. This classification is crucial for analyzing how adolescent responses differ from adult patterns.

### Measures

**The Patient Health Questionnaire-9 (PHQ-9)** was used as a validated instrument for assessing Depressive Disorder (DD) in adolescents. It consistently demonstrated high reliability, as evidenced by a Cronbach’s α coefficient ranging from 0.86 to 0.89. The PHQ-9’s sensitivity for evaluating depressive symptoms in adolescents was 89.5%, with a specificity of 77.5%, reflecting its performance in adult assessments [21, 22]. This instrument assesses each of the nine DSM-5 depression criteria on a scale of“0”(not at all) to“3”(nearly every day). An example item from this tool is, ‘Over the last two weeks, how often have you been bothered by little interest or pleasure in doing things?’

**The Generalized Anxiety Disorder 7-item scale (GAD-7)** was a reliable measure for assessing anxiety symptoms in adolescents. A GAD-7 score of 10 or above demonstrated a sensitivity of 89% and a specificity of 82% for diagnosing Generalized Anxiety Disorder during psychiatric evaluations [23]. It effectively detected clinically significant anxiety symptoms in the adolescent population [24].The Chinese version of the GAD-7 exhibited commendable internal consistency, with a Cronbach’s α coefficient ranging from 0.93 to 0.95 [25]. An example item could be, “Over the last two weeks, how often have you been bothered by feeling nervous, anxious, or on edge?”

**The Patient Health Questionnaire-15 (PHQ-15)** was employed as an efficacious instrument for evaluating somatic symptoms, demonstrating a sensitivity of 80.2% and a specificity of 58.5% [26, 27].The Chinese version of the PHQ-15 exhibited commendable internal consistency, with a Cronbach’s α coefficient of 0.83 [28]. A sample item from this tool might be, ‘In the last four weeks, how much have you been bothered by stomach pain?’

### Statistical analysis

For this study, encompassing both binary and continuous variables, demographic characteristics across groups were analyzed using the Chi-square test for categorical variables and the Bonferroni correction for continuous measures. The Chi-square test assessed the distribution ratios of blunted Niacin Response and normal Niacin Response among individuals with adolescent DD, BED, and HC to evaluate the degree of NSFR impairment across different groups. The Mann-Whitney U test and Fisher’s Exact Test compared demographic features within the two adolescent DD subgroups. Parameters were estimated using the nonlinear least squares method. Pearson correlation analysis determined the relationships between NSFR and the severities of anxiety and depression in adolescent Depressive disorder (DD) patients. Receiver Operating Characteristic (ROC) curve analyses were then applied to gauge the efficacy of combined NSFR and symptom scores in differentiating between adolescent DD and BED. All statistical analyses were performed using R studio (Version 4.1.2). SPSS 27 and GraphPad Prism 8 with a P value of less than 0.05 denoting statistical significance and all probabilities calculated as two-tailed. In this research, we utilized the GPT-4 model for assisted writing, aiming to improve the logical structure and language expression of the paper. It is noteworthy that the entire generation process took place under the careful supervision of the authors.

## Results

### Demographic characteristics

A total of 196 participants, aged between 12 and 18, were enrolled in this study.The mean age of the participants was 14.72 years, with females constituting 73.5% of the sample. Detailed data on BMI, Gender, smoking, drinking, and self-harm rates were presented in Table 1. No significant differences were observed in gender, height, weight, or BMI between the adolescent DD, BED and HC group (all *p* > 0.05). However, a statistically significant difference was noted in age between the groups (*p* < 0.05). Adolescent DD patients comprised 48.4% in junior high and 51.6% in high school. For those with BED, 68.8% were in junior high and 31.3% were in high school. All HCs were in their first year of high school. Furthermore, 37.5% of the adolescent DD patients had a family history of mental illness, 16.4% showed psychotic symptoms, 21.9% had comorbid anxiety disorders, and 2.3% had comorbid obsessive-compulsive disorder. Notably, 10.2% did not have thoughts of self-harm or suicide, whereas 89.8% did in the adolescent DD group. In the BED group, 43.8% had a family history of mental illness, 25% did not have self-harm or suicidal thoughts, and 75% did. Before admission, 63.1% of adolescent DD and BED were already undergoing treatment with selective serotonin reuptake inhibitors (SSRIs) for depression. The detailed information is delineated in Table 1.

**Table 1:** Demographic characteristics of the study sample

	DD	BED	HC	p-Value
Total number	128	32	36	-
Age (year, mean ± SD)*	14.69 ± 1.76	13.94 ± 1.46	15.52 ± 0.51	0.00^b^
Male/female	32/96	10/22	10/26	0.76^a^
Height (m, mean ± SD)	164.30 ± 7.84	163.94 ± 9.09	166.83 ± 9.34	0.24^b^
Weight (kg, mean ± SD)	56.24 ± 13.05	58.17 ± 58.17	58.0 ± 12.24	0.68^b^
BMI (kg/m^2^, mean ± SD)	20.69 ± 3.72	21.34 ± 4.96	20.61 ± 2.76	0.65^b^
Smoking(Yes, No)	15/113	2/30	0/36	0.076^a^
Drinking(Yes, No)	15/113	1/31	0/36	0.04^a^
Antidepressants prior to admission(Yes, No)*	84/44	17/15	0/36	0.00^a^
Self-harm(Yes, No)*	84/44	15/17	2/34	0.00^a^
PHQ-9(mean ± SD)*	18.68 ± 5.23	14.71 ± 6.51	4.89 ± 2.35	0.00^b^
GAD-7(mean ± SD)*	13.57 ± 4.40	12.00 ± 5.41	5.43 ± 3.96	0.00^b^
PHQ-15(mean ± SD)*	14.53 ± 5.64	12.06 ± 12.06	6.77 ± 3.99	0.00^b^

*Note* DD: Adolescence Depressive Disorder; BED: Behavioral and Emotional Disorders typically emerging in childhood and adolescence; HC: Healthy Control; SD: Standard Deviation; BMI: Body Mass Index. ^a^ Chi-square analysis. ^b^ Oneway ANOVA.

*p < 0.05

### Increased prevalence of severe blunted niacin skin flush response in adolescent DD

**Fig. 1:**
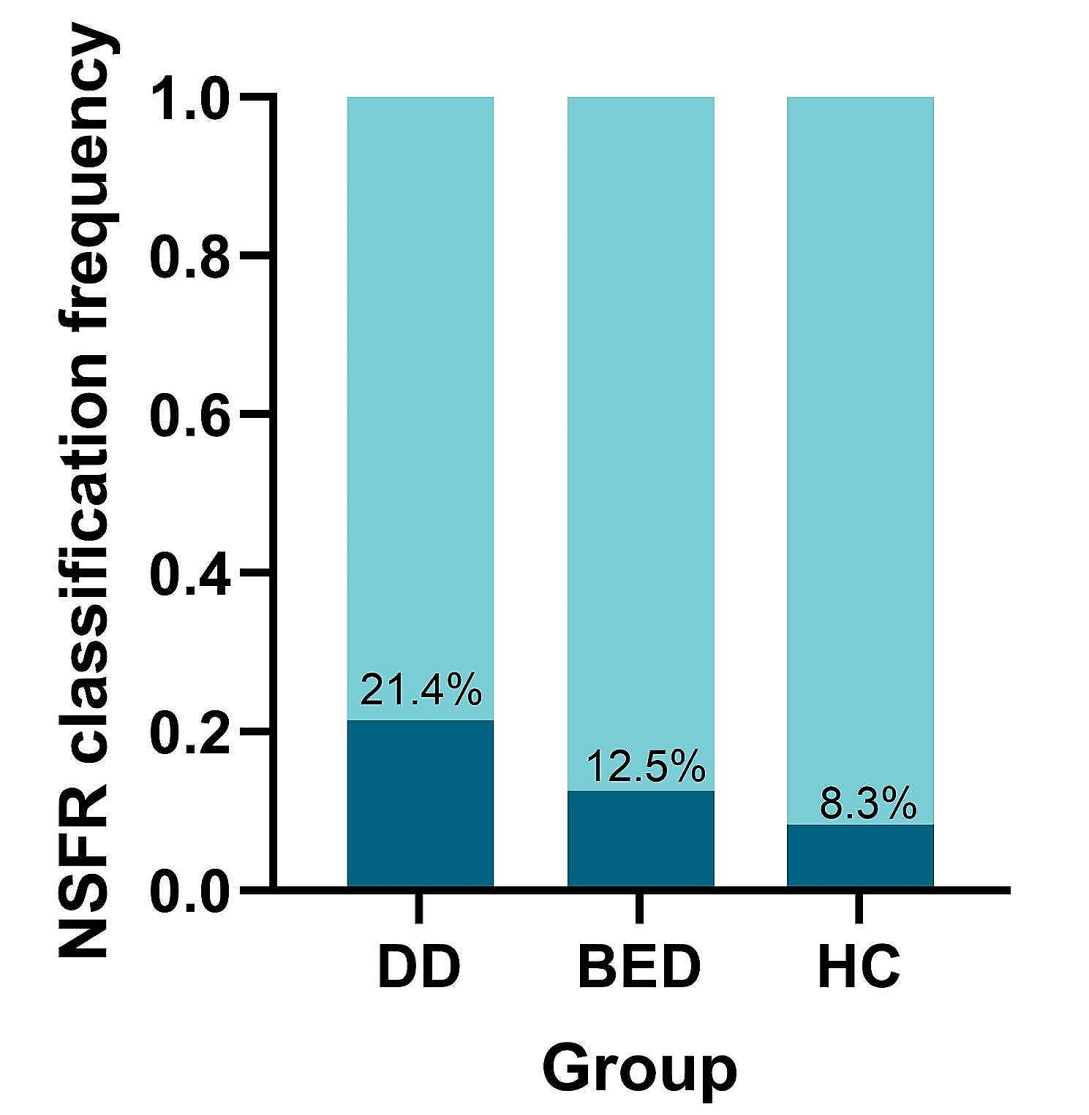
Distribution Proportion of NMB and MSB among Different Groups. Dark blue indicates the population with the normal to mild blunted NSFR, while light blue indicates the population with the moderate to severe blunted NSFR. Abbreviations: DD: Adolescence Depressive Disorder; BED: Behavioral and Emotional Disorders typically emerging in childhood and adolescence; HC: Healthy control; NSFR: Niacin Skin Flush Reaction, NMB: the normal to mild blunted NSFR, MSB: moderate to severe bunted NSFR.

In order to assess variations in NSFR across different disease cohorts and the healthy control (HC) group, chi-square tests were employed to analyze the distribution of normal to mild blunted NSFR (NMB) and moderate to severe blunted NSFR (MSB) within diverse subject groups. Comparative analysis with the BED group revealed a higher incidence of MSB subjects in adolescent DD group (21.4%) as opposed to the BED group (12.5%), with the HC group showing the lowest representation (8.3%) (refer to Fig. 1). While the chi-square test did not reach statistical significance (*p* = 0.136), our findings suggest an elevated proportion of moderate to severe blunted NSFR in the DD group compared to the HC group.

### Utilizing NSFR for subgroup classification in adolescent DD

**Fig. 2:**
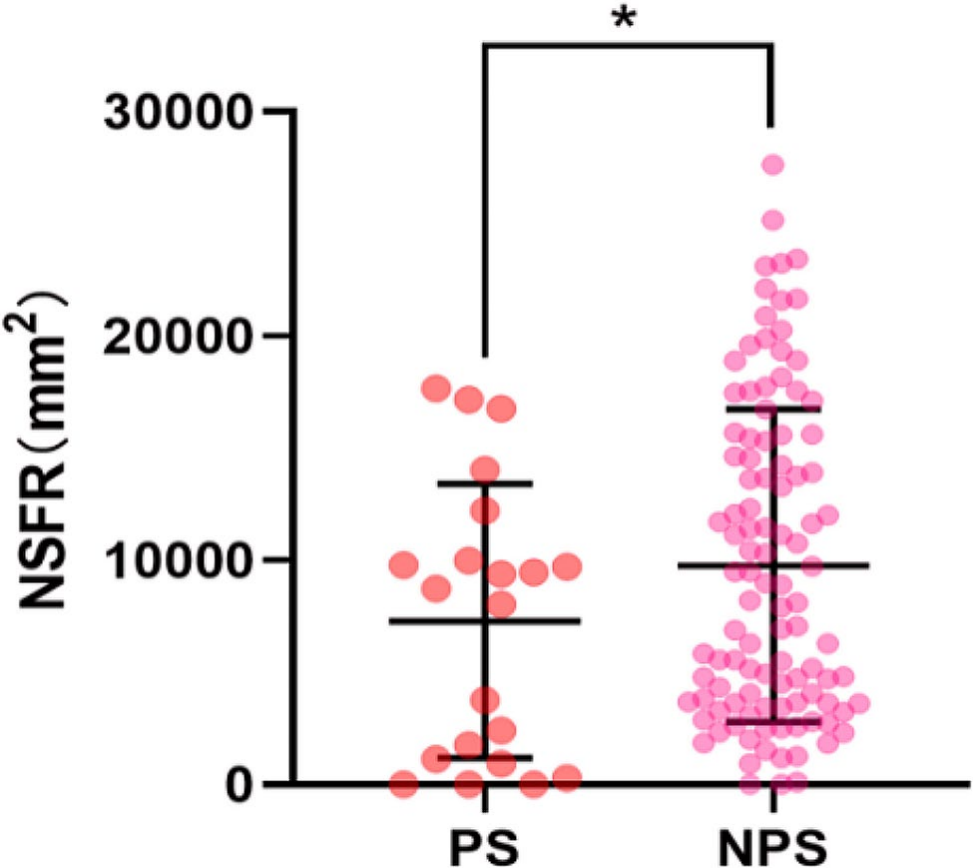
Heightened Blunted NSFR in DD with Psychotic Symptoms. Heightened blunted NSFR in adolescent DD with Psychotic Symptoms(PS) vs.Non-Psychotic Symptoms (NPS) (*p* = 0.016). NSFR: Niacin Skin Flush Reaction

Patients with Depressive Disorder (DD) were divided into two subgroups: those with and those without psychotic symptoms. Using Fisher’s Exact Test and the Mann-Whitney U test for comparative analysis, no significant differences were found between the groups in terms of gender, age, weight, height, or Body Mass Index (BMI), as well as the severity of depressive (PHQ-9) and somatic symptoms (PHQ-15). However, anxiety levels (GAD-7) were higher in the subgroup with psychotic features. This subgroup also showed a significantly heightened blunted NSFR (*p* = 0.016) when compared to those without psychotic symptoms (Fig. 2).

### NSFR assists in the differential diagnosis of adolescent DD

**Fig. 3:**
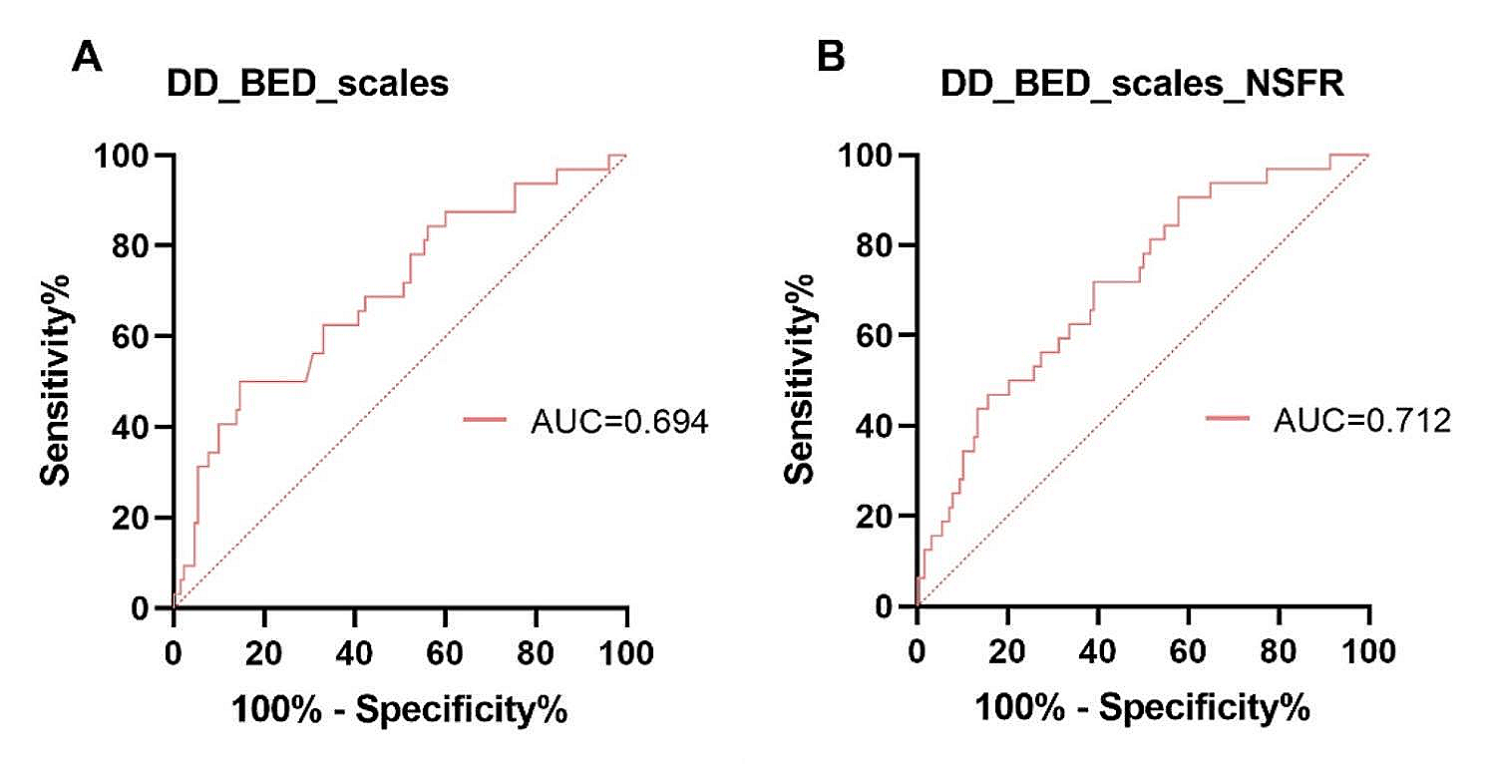
NSFR and Scales Scoring Assisted Diagnosis of adolescent DD and BED.Biomarker for identification of adolescent DD and BED via niacin skin flushing response. ROC curves of the niacin-flushing degree for distinguishing DD from BED increased from 0.694 to 0.712 (Fig. 3A&B). ROC: Receiver Operating Characteristic; AUC: Area Under Curve. NSFR: Niacin Skin Flush Reaction

The ability of NSFR to distinguish between adolescent Depressive Disorder (DD) and Behavioral and Emotional Disorders (BED) was evaluated using a focused diagnostic model. This model, employing binary logistic regression with scale and NSFR data, aimed to differentiate DD from BED. Given the small sample size for BED, additional segmentation analysis is recommended. When only scale data were used, the diagnostic accuracy was modest, with an AUC of 0.694, which underscores the clinical symptoms’ overlap. The incorporation of NSFR data enhanced the diagnostic precision, elevating the AUC to 0.712 as depicted in Fig. 3A&B. This increase demonstrates the effectiveness of NSFR in specifically distinguishing DD from BED.

### NSFR exhibits strong association with clinical symptoms in adolescent DD

**Fig. 4:**
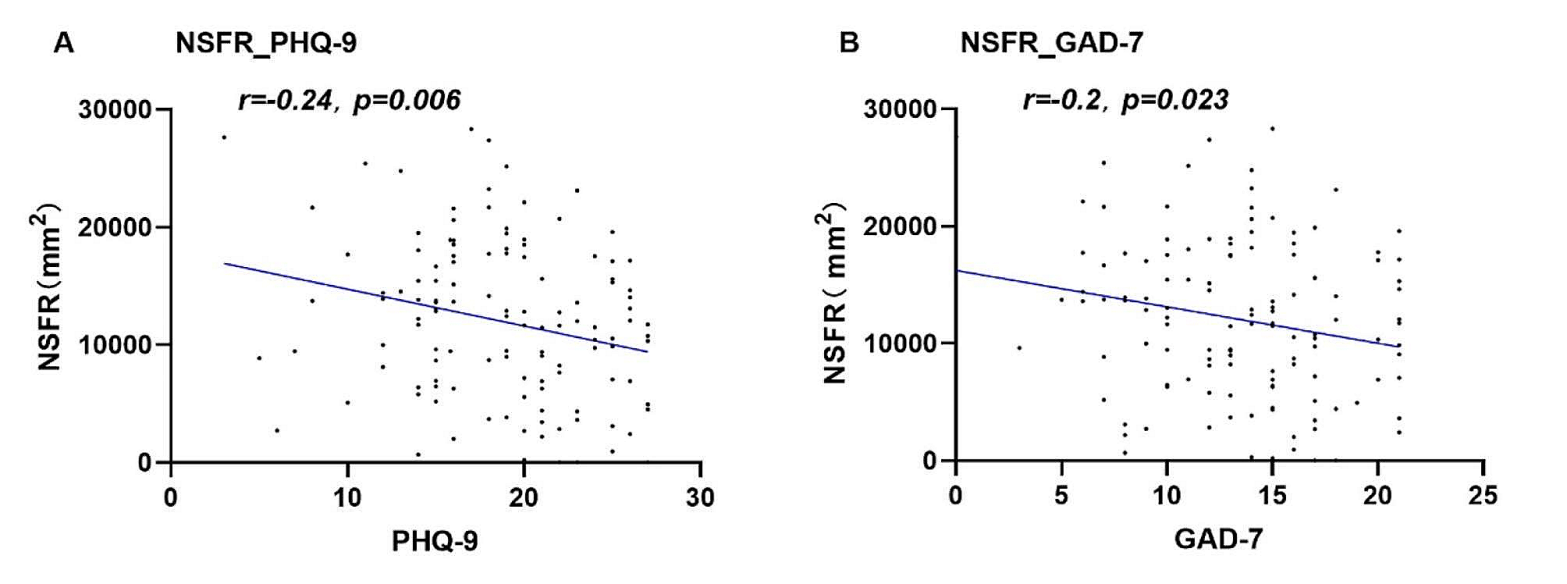
Negative Correlation of NSFR with PHQ9 and GAD7 in adolescent DD. Note: The graph demonstrates that an increase in the severity of these symptoms correlates with a more pronounced blunting of Niacin Skin Flush Reaction (NSFR)

Pearson correlation analysis was conducted to investigate the relationship between Niacin Skin Flush Response (NSFR) and symptoms of depression, anxiety, and somatization. The findings revealed a notable negative correlation between NSFR and the severity of depressive symptoms (as measured by PHQ-9) in the DD group (*R*=-0.240, *p* = 0.006). Similarly, a significant negative association was identified between NSFR and anxiety severity (as assessed by GAD-7) (*R*=-0.200, *p* = 0.023) (Fig. 4). However, no significant relationship was detected with somatization symptoms (*R*=-0.160, *p* = 0.072). Intriguingly, these correlations were not observed in either the BED or HC group. This suggests a plausible hypothesis that the niacin acid phenotype of DD patients is uniquely influenced by their clinical symptoms.

## Discussion

This study represents a pioneering effort to validate the Niacin Skin Flush Response (NSFR) as an objective biomarker for the precision diagnosis of adolescent Depressive Disorder (DD). Our primary findings revealed a higher prevalence of moderate to severe blunted NSFR among DD patients, with a heightened blunted NSFR in DD patients with psychotic symptoms. Furthermore, Our results revealed that an increase in the severity of depressive and anxious symptoms correlates with a more pronounced blunting of NSFR in adolescent DD group. Notably, the integration of NSFR with established clinical scales significantly improved the accuracy of differentiating DD from BED in adolescence.

Our study contributes to understanding NSFR as a potential biomarker for adolescent DD. It reveals a notably higher prevalence of moderate to severe blunted NSFR in DD (21.4%) compared to BED (12.5%) and healthy controls (8.3%). This result aligns with findings from Jinfeng Wang et al., who observed significant blunting and delay in NSFR in adolescent depression. Additionally, our study confirms the diagnostic value of NSFR. This is evidenced by AUC values of 0.719 and 0.721, demonstrating its high sensitivity in detecting adolescent DD [16]. Additionally, Qing et al.‘s work, which showed blunted NSFR across a spectrum of pediatric psychiatric disorders, further supports the biomarker’s broad applicability [15]. However, contrary to existing literature, our results did not confirm delays in NSFR typically associated with Depression, suggesting variability in NSFR response or potential age-related factors affecting the biomarker’s performance. While the proportion of blunted NSFR was higher in adolescent DD group, the lack of statistical significance necessitates cautious interpretation and underscores the need for further research with larger cohorts to validate these findings.

Our exploratory study represents the first to document a heightened blunted NSFR in adolescent DD patients with psychotic symptoms compared to those with non-psychotic symptoms. Clinically, psychotic features in depression introduce specific challenges, including a higher rate of recurrence, an elevated risk of suicide, and a potential increase in hospital admissions [29–31]. Pathophysiologically, a spectrum of susceptibility genes such as BDNF, DBH, DTNBP1, DRD2, DRD4, GSK-3beta, and MAO-A has been implicated [32]. Additionally, heightened HPA axis activity [33], and changes in the advanced associative regions of the frontal and insular cortices have been associated with an increased risk of psychotic manifestations in depression [34]. Our findings contribute a novel insight into the pathophysiological mechanisms underlying adolescent DD with psychotic symptoms. While our data indicate that NSFR maybe could assist in differentiating DD subtypes, these findings warrant cautious interpretation. The potential of NSFR as a non-invasive biomarker for stratifying patients according to psychotic symptomatology requires further validation.

Differentiating between adolescent DD and BED is a significant challenge in clinical practice [35, 36]. When we combine the niacin phenotype with clinical symptoms, the differentiation improves. These symptoms are captured by PHQ-9, GAD-7, and PHQ-15 scales. This approach is more effective than using the scales alone. Using NSFR as a biological marker may further enhance the precision in distinguishing between DD and BED in adolescents.

This groundbreaking study has unveiled a novel negative correlation between the Niacin Skin Flush Response (NSFR) and the severity of depressive and anxiety symptoms as measured by the PHQ-9 and GAD-7 scales in adolescent Depressive Disorder (DD). Our results revealed that an increase in the severity of these symptoms correlates with a more pronounced blunting of NSFR. This finding is consistent with earlier studies [14].Previous research noted a significant link between reduced NSFR and depression symptoms. It also associated diminished NSFR with anxiety symptoms in adults. Furthermore, the research connected reduced NSFR with somatic complaints. These complaints include appetite loss and weight reduction in adults.These findings highlight the potential of NSFR as a valuable tool for assessing the severity of adolescent DD.

However, as indicated in Fig. 4, we observed considerable variability in NSFR among individuals with similar levels of depressive severity. This variability could stem from the inherent heterogeneity in the etiology and clinical manifestations of adolescent DD, influenced by an interplay of biological and psychosocial factors [6, 7]. Therefore, we postulate the presence of a distinct adolescent DD subgroup characterized by a significant phospholipid signaling defect, evident as abnormal NSFR responses [14]. Such a finding could offer new perspectives on the pathophysiological underpinnings of adolescent DD. It could also inform targeted therapeutic interventions, such as the application of Omega-3 fatty acids in depression management [37].

The Niacin Skin Flush Response (NSFR) is mediated by the PLA2-AA-COX2 pathway and its prostaglandin products [16]. Increased oxidative stress has been observed in Childhood and Adolescence with DD. This leads to enhanced PLA2 activity, resulting in the excessive breakdown of membrane phospholipids [17].. This process likely contributes to the diminished NSFR observed in these individuals. Furthermore, a significant decrease in free arachidonic acid (AA) levels in the red blood cell membranes of DD in childhood and adolescence has been reported [38, 39], suggesting an AA turnover imbalance. These factors are associated with chronic inflammation. They may drive abnormal PLA2-AA-COX2 activity, culminating in niacin response blunting in DD.

### Implications

Our study highlights the potential of NSFR in improving the identification and treatment of adolescent Depressive Disorder. Transcending traditional methods, NSFR provides an innovative and objective screening tool within schools, allowing for the rapid identification of depressive conditions among middle and high school students. This approach enhances detection accuracy and increases the rate of early diagnosis, significantly reducing the reliance on subjective self-reported assessments. Additionally, in clinical settings, NSFR provides an objective evaluation of the severity of depression in adolescents. Moreover, identifying adolescent DD patients with blunted NSFR responses could guide the targeted use of Omega-3 fatty acids in managing depression, suggesting a personalized approach to treatment.

### Limitations

In our study’s limitations, the significant disparity in group size might have affected the statistical outcomes, including Cohen’s d values. Future research should ensure balanced group sizes to robustly validate NSFR’s role as a biomarker in adolescent DD. Additionally, with over half of the patients undergoing SSRI antidepressant treatment during NSFR assessment, it is imperative for subsequent studies to explore how these medications might affect NSFR results. Future research should focus on evaluating NSFR’s specificity and sensitivity in adolescent Depressive Disorder (DD) to establish its efficacy as a biological marker. Investigating NSFR’s role in longitudinal studies to monitor depressive disorder progression and its correlation with treatment outcomes is crucial. Expanding research to various populations will help determine NSFR’s generalizability as a biomarker. Additionally, exploring the molecular mechanisms linking NSFR to depression can provide deeper insights into its pathophysiology, enhancing our understanding and treatment of adolescent DD.

## Conclusion

The NSFR maybe emerge as a promising objective biological marker for screening, severity assessment, subgroup classification, and differential diagnosis in adolescent DD. It unveils novel insights into the pathophysiological underpinnings of the disorder and may lead to advancements in precision diagnosis. However, the establishment of NSFR’s clinical utility necessitates further validation through rigorous studies. These should aim to determine the specificity and sensitivity of NSFR in the context of adolescent DD and evaluate its potential as a longitudinal measure of treatment efficacy (See Table 2 for detailed abbreviations.)

## Data Availability

Availability of data and materialsThe datasets generated during the current study are not publicly available due to the subjects’ privacy but are available from the corresponding author on reasonable request.

## Abbreviations

DD: Depressive Disorder
BED: Behavioral and Emotional Disorders typically emerging in childhood and adolescence
HC: Healthy control
PLA2: Phospholipase A2
AA: Arachidonic acid
COX2: Cyclooxygenase 2
AMN: Aqueous methyl nicotinate
SSRI: Selective serotonin reuptake inhibitor
NSFR: Niacin Skin Flush Reaction
NMB: the normal to mild blunted NSFR
MSB: moderate to severe bunted NSFR
PS: DD with Psychotic Symptom
NPS: Non-Psychotic Symptoms
ROC: Receiver Operating Characteristic
AUC: Area Under Curve
CIDI: Composite International Diagnostic Interview
